# PM2.5 Exposure Disrupts Sympathetic Neurotransmission by Impairing Voltage-Gated Ca^2+^ Channels and Remodeling Satellite Glia in the Superior Cervical Ganglion

**DOI:** 10.3390/ijms27209184

**Published:** 2026-10-16

**Authors:** Phuong Anh Do, Huu Son Nguyen, Seung-Kuy Cha

**Affiliations:** 1 Department of Physiology, Yonsei University Wonju College of Medicine, Wonju 26426, Republic of Korea; phuonganh3110dkh@gmail.com (P.A.D.); son.nh3@vinuni.edu.vn (H.S.N.); 2 Department of Global Medical Science, Yonsei University Wonju College of Medicine, Wonju 26426, Republic of Korea; 3 Organelle Medicine Research Center, Yonsei University Wonju College of Medicine, Wonju 26426, Republic of Korea; 4 Vinmec Research Institute of Stem Cell and Gene Technology, College of Health Sciences, VinUniversity, Hanoi 100000, Vietnam; 5 Institute of Mitochondrial Medicine, Yonsei University Wonju College of Medicine, Wonju 26426, Republic of Korea

**Keywords:** particulate matter, superior cervical ganglion, satellite glial cells, sympathetic nervous system, voltage-gated calcium channels

## Abstract

Airborne fine particulate matter (PM2.5) exposure is associated with central neuroinflammation and autonomic dysfunction, but its effects on peripheral sympathetic ganglia remain poorly defined. Here, we examined the effects of central PM2.5 administration and direct PM2.5 exposure on the superior cervical ganglion (SCG), a primary sympathetic relay governing cephalic noradrenergic outflow. Intracerebroventricular (ICV) administration of PM2.5 increased hypothalamic TNF-α and IL-6 levels and reduced presynaptic synaptophysin expression, while SCG tyrosine hydroxylase expression remained unchanged. In a complementary ex vivo model, direct PM2.5 exposure reduced norepinephrine (NE) accumulation in the culture medium of isolated SCGs without significantly altering tissue NE content. Mechanistically, live-cell Ca^2+^ imaging revealed attenuated depolarization-evoked Ca^2+^ responses in SCG neurons following either 24-h direct exposure or in vivo ICV administration. Whole-cell patch-clamp recordings further demonstrated reduced voltage-gated Ca^2+^ channel (VGCC) current density in neurons isolated from ICV PM2.5-treated rats. This functional reduction was not accompanied by significant changes in the SCG mRNA levels of the examined pore-forming Ca_v_α1 subunits. In parallel, ICV PM2.5 administration increased GFAP and Kir4.1 protein levels and enhanced perineuronal GFAP immunoreactivity, consistent with satellite glial cell reactivity. Together, these findings identify impaired neuronal Ca^2+^ signaling and accompanying glial alterations in the SCG following PM2.5 exposure. The reductions in extracellular NE accumulation and VGCC function across complementary exposure models support a potential contribution of impaired Ca^2+^-dependent secretion to PM2.5-associated sympathetic dysfunction.

## 1. Introduction

Air pollution driven by ambient fine particulate matter with an aerodynamic diameter ≤ 2.5 µm (particulate matter ≤ 2.5 µm; PM2.5) is widely recognized as an environmental driver of systemic cardiopulmonary morbidity and autonomic dysregulation. Beyond respiratory pathology, growing evidence highlights the central nervous system (CNS) as a critical target of airborne pollutants [1,2]. Inhaled particulate matter can penetrate the brain parenchyma—predominantly via olfactory transmucosal translocation or blood–brain barrier compromise—eliciting marked neuroinflammation, microglial activation, and neurotoxic cascades [1,2,3,4]. Among vulnerable brain regions, the hypothalamus is especially pivotal because it centrally controls neuroendocrine and autonomic homeostatic networks. Our previous investigations demonstrated that PM2.5 triggers profound hypothalamic oxidative stress, NOX4 upregulation, and endoplasmic reticulum stress, ultimately culminating in synaptic and neuronal vulnerability [1,4]. Both acute and chronic exposure to concentrated ambient particles has been shown to induce hypothalamic inflammation coupled with elevated arterial pressure and heightened sympathetic tone, an effect reversible with central sympathetic blockade [1,5,6]. These observations strongly implicate particulate matter-induced central neuropathology in downstream autonomic imbalance.

However, the current understanding of PM2.5-associated sympathetic dysfunction relies almost exclusively on macroscopic, integrated endpoints, such as elevated resting blood pressure, blunted heart rate variability, or elevated circulating and urinary catecholamines [6,7,8,9]. While informative, these composite physiological indices fail to resolve the distinct cellular loci of autonomic failure—specifically conflating central premotor drive with the intrinsic functional fidelity of peripheral sympathetic relay ganglia. Sympathetic ganglia are not merely passive conduits; they serve as specialized integrating stations that connect central descending preganglionic inputs from the spinal cord intermediolateral nucleus (IML) to target-innervating postganglionic effectors [10,11]. Consequently, descending neuroinflammatory signals or direct peripheral exposure could profoundly alter the cellular excitability and secretory competence of sympathetic ganglionic neurons. Yet, whether PM2.5 exposure impairs peripheral sympathetic ganglion physiology remains fundamentally uncharted.

The superior cervical ganglion (SCG), the rostral relay of the sympathetic paravertebral chain, offers a well-established model to investigate peripheral sympathetic neurotransmission [12]. Postganglionic noradrenergic neurons in the SCG govern cephalic and cervicofacial targets [12], where vesicular norepinephrine (NE) exocytosis is strictly governed by depolarization-evoked Ca^2+^ influx through voltage-gated Ca^2+^ channels (VGCCs) [13,14].

Disruptions in VGCC function can directly decouple electrical activation from chemical transmission, regardless of catecholaminergic synthesis or storage capacity. Furthermore, satellite glial cells (SGCs) intimately wrap autonomic somata, forming tightly organized neuron-SGC functional units [15]. By orchestrating extracellular K^+^ buffering via inward rectifier K^+^ channels such as Kir4.1 and modulating neurochemical dynamics, SGCs actively govern local neuronal excitability and display prominent reactive transformation during inflammatory insults [16,17]. Thus, determining the peripheral neurotoxicity of PM2.5 requires examining both intrinsic neuronal channel gating and the surrounding glial microenvironment.

In this study, we combined intracerebroventricular (ICV) PM2.5 administration with direct exposure of isolated SCG and cultured SCG neurons to investigate PM2.5 effects on peripheral sympathetic ganglion function. We found that central PM2.5 exposure was associated with hypothalamic inflammation, reduced neuronal VGCC current density, and reactive SGC changes accompanied by increased Kir4.1 expression in the SCG. Together, these findings identify the SCG as a potential peripheral target of PM2.5 toxicity and support impaired neuronal Ca^2+^ signaling, alongside glial remodeling, as a potential contributor to PM2.5-associated sympathetic dysfunction.

## 2. Results

### 2.1. Intracerebroventricular PM2.5 Exposure Induces Hypothalamic Neuroinflammation and Synaptic Impairment

**Figure 1:**
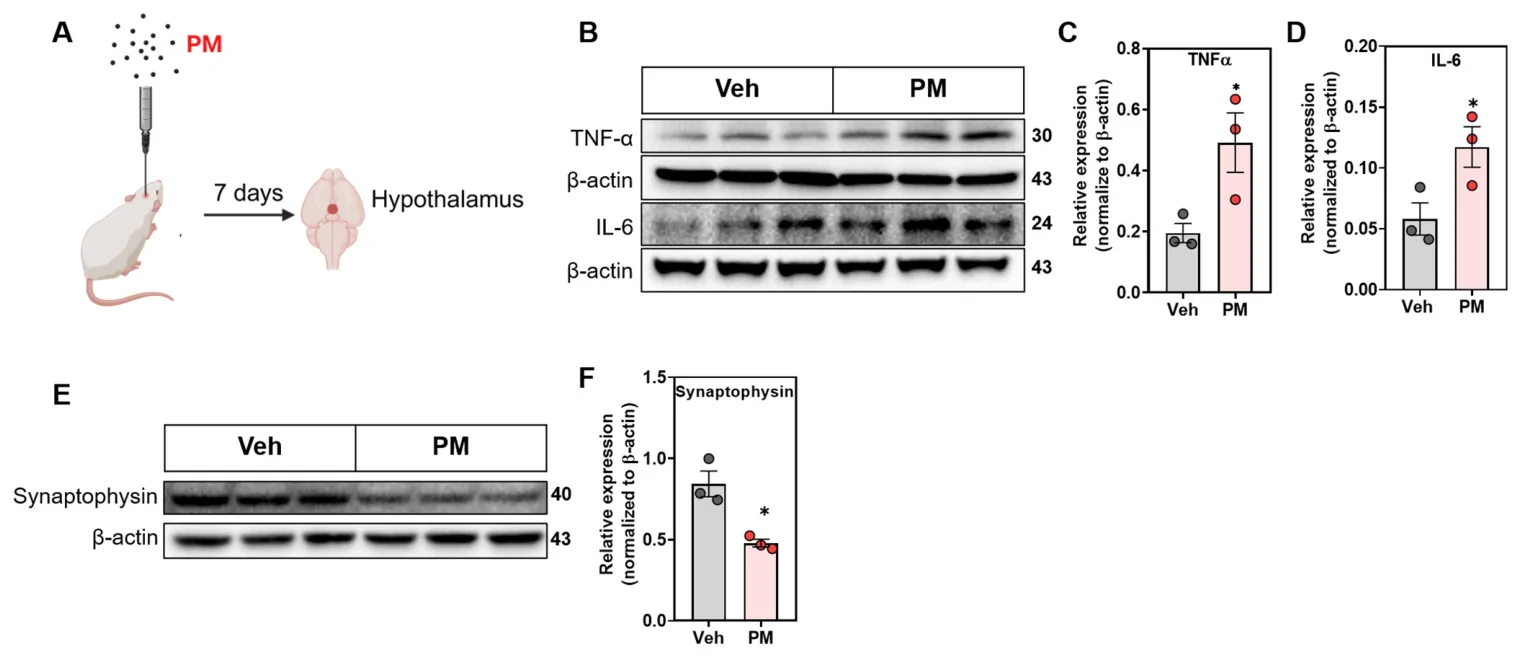
PM2.5-induced hypothalamic inflammation. (**A**) Experimental design for PM2.5 stereotaxic injection and hypothalamus collection. (**B**) Immunoblot analysis showing inflammation marker expression in the hypothalamus after PM2.5 treatment. β-actin was used as a loading control. (**C**) Quantification of TNF-α levels (*n* = 3 rats per group). (**D**) Quantification of IL-6 levels (*n* = 3 rats per group). (**E**) Immunoblot analysis showing synaptophysin expression in the hypothalamus after PM2.5 treatment. β-actin was used as a loading control. (**F**) Quantification of synaptophysin levels (*n* = 3 rats per group). Data are presented as mean ± SEM. * *p* ≤ 0.05 versus the vehicle-treated group.

Previous studies, including our own, have demonstrated that particulate matter (PM2.5) can translocate into the brain via the olfactory pathway and exert neurotoxic effects, with prominent effects in the hypothalamus [1,2,18,19,20]. To directly test whether central PM2.5-induced neuroinflammation is linked to downstream sympathetic dysregulation while bypassing potential systemic confounders, we established an intracerebroventricular (ICV) PM2.5 exposure model in rats using stereotaxic delivery (Figure 1A, Supplementary Figure S1).

Seven days post-injection, Western blot analysis revealed marked increases in hypothalamic pro-inflammatory cytokines: both TNF-α and IL-6 protein levels were significantly elevated in the PM2.5-treated group compared to vehicle controls (Figure 1B–D), confirming sustained local neuroinflammation. We also examined hypothalamic synaptic integrity by assessing synaptophysin, a key presynaptic vesicle marker. Consistent with neuroinflammatory injury, hypothalamic synaptophysin expression was significantly decreased in PM2.5-treated rats relative to the vehicle groups (Figure 1E,F).

Together, these findings indicate that central PM2.5 exposure establishes a persistent neuroinflammatory milieu and compromises presynaptic integrity within the hypothalamus, setting the stage for investigating secondary peripheral sympathetic signaling alterations.

### 2.2. Effect of PM2.5 on SCG Tyrosine Hydroxylase Expression In Vivo and Extracellular Norepinephrine Accumulation Ex Vivo

**Figure 2:**
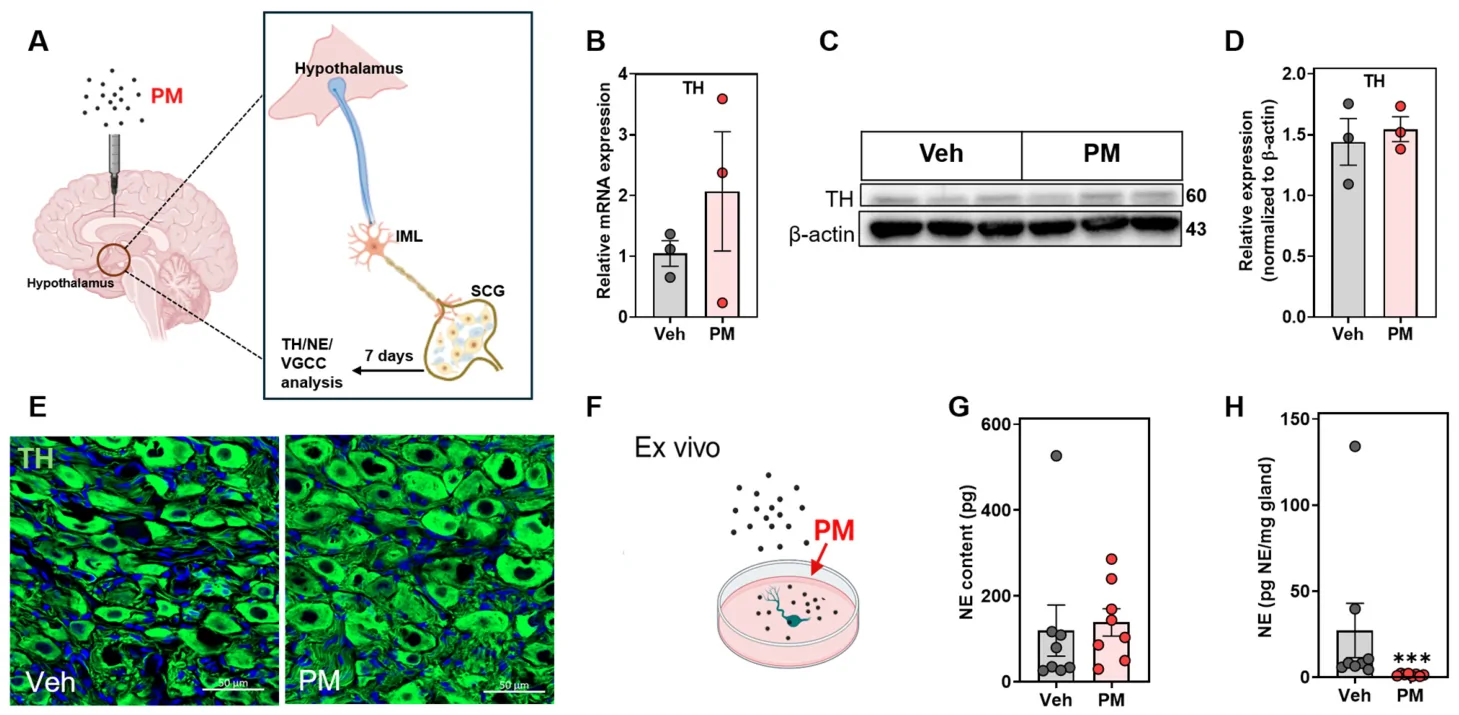
Effects of PM2.5 on SCG TH expression in vivo and NE accumulation ex vivo. (**A**) Schematic illustration of the hypothalamic–sympathetic pathway to the SCG and experimental design for PM2.5 stereotaxic injection and SCG collection. (**B**) Quantification of TH mRNA levels (*n* = 3 rats per group). (**C**) Immunoblot analysis showing TH expression in SCG after PM2.5 treatment. β-actin was used as a loading control. (**D**) Quantification of TH protein levels (*n* = 3 rats per group). (**E**) Immunostaining analysis showing TH expression in SCG after PM2.5 treatment. (**F**) Experimental design for ex vivo experiments in SCG. (**G**,**H**) NE content in SCG tissue (**G**) and secreted NE in the cultured media (**H**), measured by ELISA, in PM2.5-treated groups compared with vehicle-treated groups (*n* = 8 rats per group). Data are presented as mean ± SEM. *** *p* ≤ 0.001 versus the vehicle-treated group.

Central sympathetic outflow originates in the hypothalamus and descends through the spinal cord intermediolateral nucleus (IML) to activate the superior cervical ganglion (SCG), the principal rostral relay station that projects noradrenergic postganglionic fibers to peripheral target tissues (Figure 2A). Following the hypothalamic inflammatory changes observed after central PM2.5 administration, we examined tyrosine hydroxylase (TH) expression in the SCG and, in a separate ex vivo experiment, assessed the direct effects of PM2.5 on ganglionic NE content and extracellular NE accumulation.

For the in vivo experiments, SCGs were collected seven days after stereotaxic ICV administration of PM2.5 or vehicle (Figure 2A). Quantitative RT-PCR and immunoblot analyses showed no significant differences in TH mRNA or protein levels, the rate-limiting enzyme in catecholamine biosynthesis, between groups (Figure 2B–D). Consistent with these findings, confocal immunofluorescence analysis of SCG tissue sections revealed comparable TH immunoreactivity in vehicle- and PM2.5-treated rats (Figure 2E).

In a separate ex vivo experiment, freshly isolated SCGs were cultured and directly exposed to PM2.5 or vehicle for 24 h (Figure 2F). ELISA measurements showed no significant difference in tissue NE content between groups (Figure 2G), whereas NE accumulation in the culture medium was significantly reduced following PM2.5 exposure (Figure 2H). Thus, direct PM2.5 exposure reduced extracellular NE accumulation without a detectable decrease in tissue NE content.

Together, these findings demonstrate that ICV PM2.5 administration did not significantly alter SCG TH expression, whereas direct ex vivo exposure reduced extracellular NE release without significantly changing tissue NE content.

### 2.3. PM2.5 Exposure Attenuates Depolarization-Evoked Ca^2+^ Responses and Reduces VGCC Current Density in SCG Neurons

Depolarization-induced Ca^2+^ influx through voltage-gated Ca^2+^ channels (VGCCs) is a key trigger for synaptic vesicle exocytosis and NE release [21,22,23]. After observing reduced NE accumulation in PM2.5-exposed ex vivo SCG cultures (Figure 2H), we next examined PM2.5 effects on depolarization-evoked Ca^2+^ signaling and VGCC currents. We assessed the effects of direct in vitro PM2.5 exposure and examined neurons from rats after ICV PM2.5 administration.

We first examined whether acute PM2.5 application directly altered VGCC currents in isolated SCG neurons using whole-cell patch-clamp recording (Supplementary Figure S2A). A voltage ramp from −80 to +80 mV elicited voltage-dependent inward currents. Acute bath application of PM2.5 did not immediately suppress these currents under the recording conditions (Supplementary Figure S2B).

**Figure 3:**
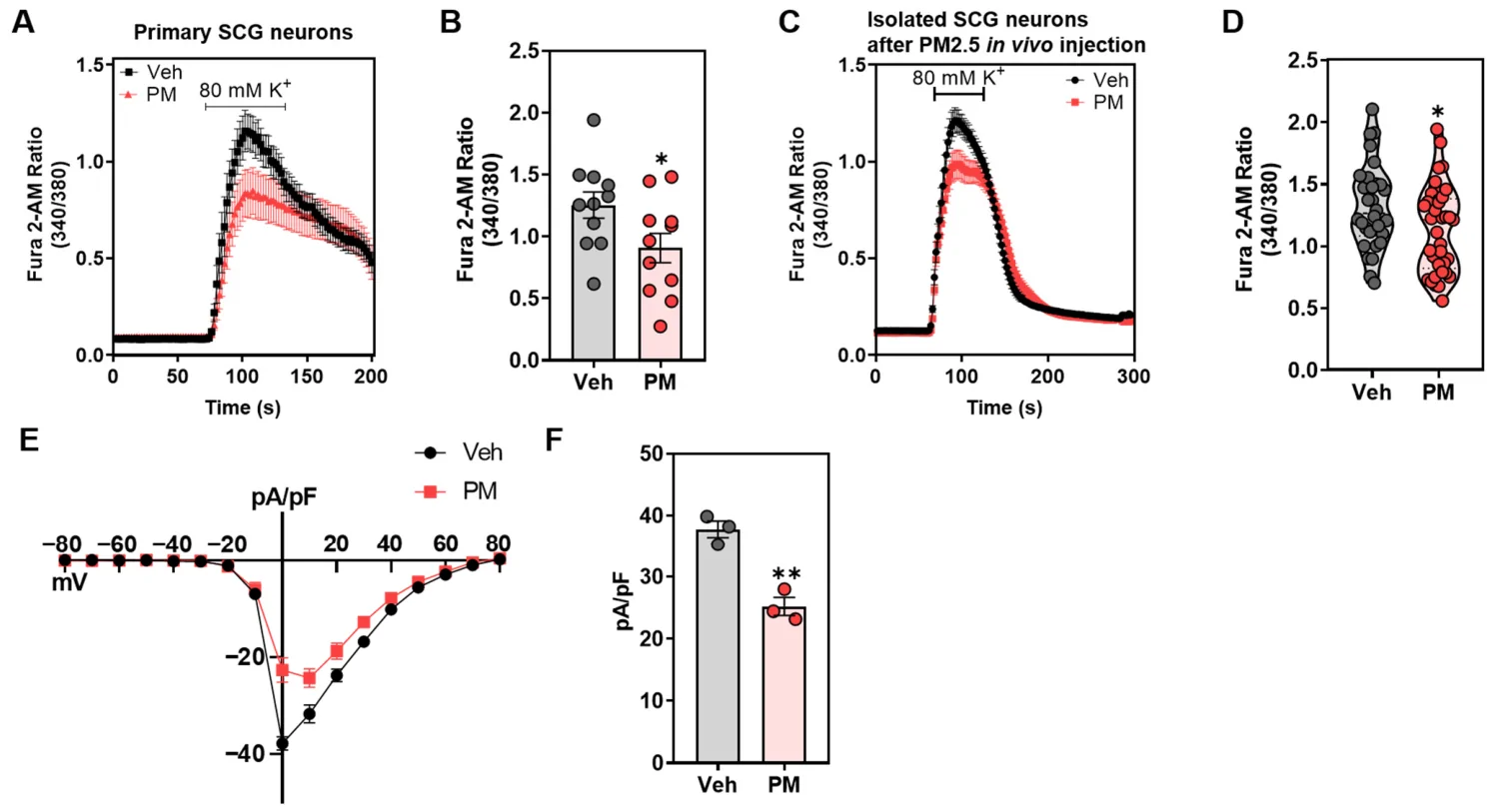
PM2.5 impairs Ca^2+^ influx via VGCCs in the SCG. (**A**) Representative traces of high VGCC activity induced by high K^+^ stimulation (80 mM K^+^) in primary SCG neurons treated with vehicle or PM2.5 (100 μg/mL) for 24 h. (**B**) Quantification of Fura-2 ratio in PM2.5-treated and vehicle-treated SCG neurons (*n* = 11 cells per group). (**C**) Representative traces of high VGCC activity induced by high K^+^ stimulation (80 mM K^+^) in primary SCG neurons isolated from vehicle- or PM2.5-injected rats. (**D**) Quantification of Fura-2 ratio in vehicle-treated and PM2.5-treated rats (*n* = 30 and 37 cells for vehicle- and PM2.5-treated groups, respectively). (**E**) Summary *I*–*V* curves for SCG neurons isolated from vehicle- or PM2.5-treated rats. (**F**) Quantification of peak VGCC current density in vehicle- or PM2.5-treated rats (*n* = 3 cells per group). Data are presented as mean ± SEM. * *p* ≤ 0.05, ** *p* ≤ 0.01 versus the vehicle-treated group.

We next assessed depolarization-evoked Ca^2+^ responses after 24 h of direct PM2.5 exposure (Supplementary Figure S3A). Primary SCG neurons were treated with PM2.5 or vehicle and subsequently loaded with Fura-2 AM. High K^+^ (80 mM K^+^) evoked intracellular Ca^2+^ ([Ca^2+^]*_i_*) transients, with a significantly reduced peak response in PM2.5-treated neurons compared with vehicle-treated controls (Figure 3A,B). To determine whether a similar deficit occurred following in vivo exposure, we examined SCG neurons isolated from rats seven days after ICV administration of PM2.5 or vehicle (Supplementary Figure S3B). Neurons from PM2.5-treated rats likewise exhibited significantly reduced depolarization-evoked peak [Ca^2+^]*_i_* responses to high-K^+^ stimulation compared with those from vehicle-treated rats (Figure 3C,D). Thus, we observed attenuated depolarization-evoked Ca^2+^ responses after both direct in vitro exposure and ICV exposure in vivo.

To assess whether the attenuated Ca^2+^ responses following in vivo exposure were accompanied by reduced VGCC currents, we performed whole-cell patch-clamp recordings in SCG neurons isolated seven days after ICV administration. Individual current traces are shown in Supplementary Figure S3C. Current–voltage (*I*–*V*) analysis revealed reduced inward current densities in neurons from PM2.5 -treated rats, with a significant decrease in peak VGCC current density compared with controls (Figure 3E,F). No significant difference in the voltage dependence of activation was detected between groups (Supplementary Figure S3D).

Taken together, these findings demonstrate attenuated depolarization-evoked Ca^2+^ responses and VGCC current density following direct or ICV PM2.5 exposure. In conjunction with the reduced extracellular NE accumulation observed in ex vivo SCG cultures, these results support a potential contribution of impaired VGCC-mediated Ca^2+^ entry to altered sympathetic secretion.

### 2.4. Reduced VGCC Function Is Not Accompanied by Significant Changes in Pore-Forming Subunit mRNA Expression Levels

**Figure 4:**
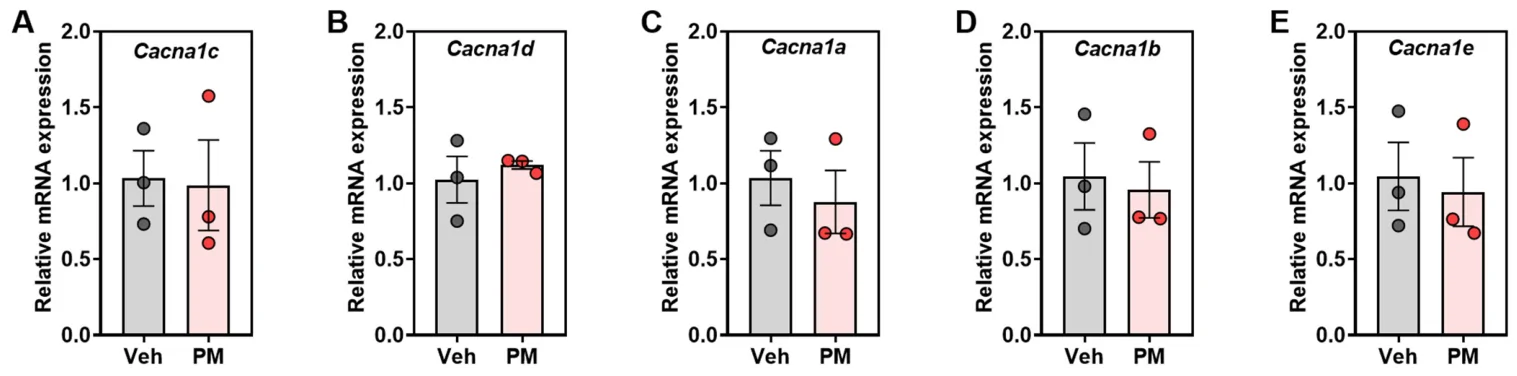
PM2.5 did not change VGCC mRNA expression. (**A**) Quantification of *Cacna1c* (encoding Ca_v_1.2α1) mRNA expression in SCG tissue following PM2.5 stereotaxic injection *(n* = 3 rats per group). (**B**) Quantification of *Cacna1d* (Ca_v_1.3α1) mRNA expression in SCG tissue following PM2.5 stereotaxic injection (*n* = 3 rats per group). (**C**) Quantification of *Cacna1a* (Ca_v_2.1α1) mRNA expression in SCG tissue following PM2.5 stereotaxic injection (*n* = 3 rats per group). (**D**) Quantification of *Cacna1b* (Ca_v_2.2α1) mRNA expression in SCG tissue following PM2.5 stereotaxic injection (*n* = 3 rats per group). (**E**) Quantification of *Cacna1e* (Ca_v_2.3α1) mRNA expression in SCG tissue following PM2.5 stereotaxic injection (*n* = 3 rats per group). Data are presented as mean ± SEM.

To determine whether the reduced VGCC current density and depolarization-evoked [Ca^2+^]*_i_* responses described above were accompanied by changes in channel transcript abundance, we quantified mRNA abundance of major pore-forming α1 subunits in SCG tissue by quantitative RT-PCR. We examined the transcripts encoding L-type (Ca_v_1.2 and Ca_v_1.3), P/Q type (Ca_v_2.1), N-type (Ca_v_2.2), and R-type (Ca_v_2.3) channels, which collectively mediate high-voltage-activated Ca^2+^ currents in autonomic neurons. We detected no statistically significant differences in the mRNA levels of these subunits between rats receiving ICV PM2.5 and vehicle controls (Figure 4A–E). Thus, the reduction in VGCC function was not accompanied by detectable changes in the abundance of the examined pore-forming subunit transcripts in SCG tissue.

### 2.5. Central PM2.5 Exposure Induces Satellite Glial Cell Reactivity in the SCG

We next examined whether the neuronal Ca^2+^ signaling deficits observed following ICV PM2.5 administration were accompanied by changes in the surrounding glial microenvironment. In peripheral sympathetic ganglia, satellite glial cells (SGCs) tightly surround neuronal somata and help maintain the perineuronal ionic and biochemical *milieu* [15,24,25]. We therefore assessed SGC reactivity and Kir4.1 protein expression in the SCG following central PM2.5 exposure.

**Figure 5:**
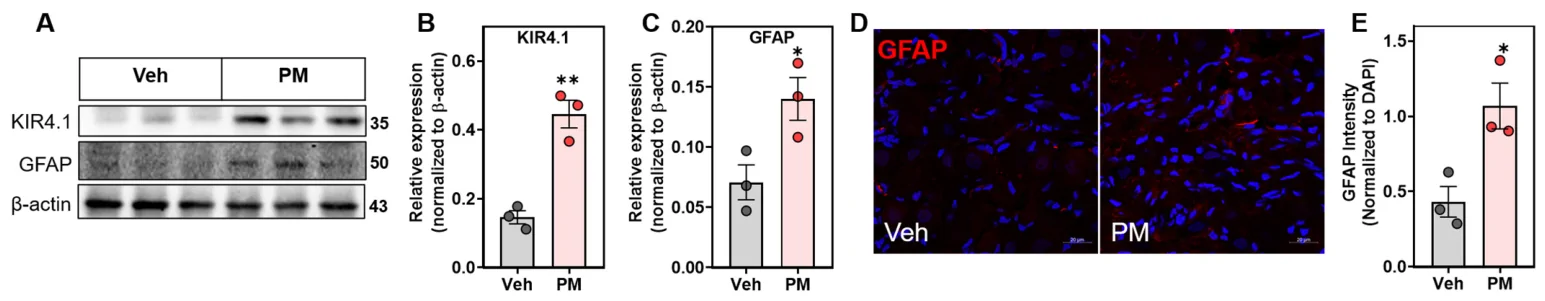
PM2.5-induced activation of SGCs in the SCG. (**A**) Immunoblot analysis showing SGC marker expression after PM2.5 stereotaxic injection. β-actin was used as a loading control. (**B**) Quantification of Kir4.1 protein levels (*n* = 3 rats per group). (**C**) Quantification of GFAP protein levels (*n* = 3 rats per group). (**D**) Immunostaining analysis showing GFAP expression in SCG after PM2.5 injection. (**E**) Quantification of GFAP levels (*n* = 3 rats per group). Data are presented as mean ± SEM. * *p* ≤ 0.05, ** *p* ≤ 0.01 versus the vehicle-treated group.

Immunoblot analysis revealed significantly increased glial fibrillary acidic protein (GFAP) levels, an established hallmark of SGC activation, in SCG tissue from PM2.5-treated rats compared with vehicle controls (Figure 5A,C). Consistent with this finding, confocal immunofluorescence imaging showed augmented GFAP immunoreactivity surrounding neuronal cell bodies (Figure 5D,E). In addition, immunoblot analysis exhibited a significant upregulation of Kir4.1 in SCG tissue following ICV PM2.5 administration (Figure 5A,B).

Together, these findings indicate that central PM2.5 exposure promotes SGC reactivity accompanied by increased Kir4.1 protein expression in the SCG. These glial alterations were observed alongside the neuronal Ca^2+^ signaling deficits described above.

## 3. Discussion

This study identified the SCG as a peripheral target of PM2.5-induced autonomic dysfunction. Using complementary exposure models, we found that ICV PM2.5 administration increased hypothalamic inflammatory markers and reduced synaptophysin expression, while SCG neurons exhibited attenuated depolarization-evoked Ca^2+^ responses and reduced VGCC current density. These changes were accompanied by increased GFAP and Kir4.1 expression in the SCG, without significant changes in TH expression or the examined VGCC pore-forming subunit transcripts. In separate direct exposure experiments, PM2.5 reduced extracellular NE accumulation in ex vivo SCG cultures without significantly changing tissue NE content and attenuated Ca^2+^ responses in cultured SCG neurons. Together, these findings support a potential contribution of impaired neuronal Ca^2+^ signaling to altered sympathetic secretion, alongside reactive glial changes.

The hypothalamic changes are consistent with previous studies identifying this region as a target of PM2.5 in autonomic regulation. Prolonged exposure induces hypothalamic inflammation together with increased blood pressure, increased low-frequency blood-pressure variability, and increased urinary NE [6]. Even short-term exposure may trigger an early inflammatory response in this region [5]. PM2.5 also impairs synaptic function through NF-κB and the miR-574-5p/BACE1 axis [26], whereas urban PM reduces synaptic responses in CA1 neurons through an NMDA/NO-dependent pathway [27]. These observations are consistent with our data showing that PM2.5 increases inflammatory markers and reduces the abundance of the presynaptic protein synaptophysin in the hypothalamus. We used our in vivo model to determine whether PM2.5-induced central alterations are accompanied by peripheral sympathetic ganglion dysfunction. To directly assess whether the SCG itself is susceptible to PM2.5, we exposed isolated SCGs to PM2.5 ex vivo, which revealed a significant reduction in extracellular NE accumulation.

Although previous reports of increased systemic sympathetic activity may appear inconsistent with the reduced extracellular NE accumulation and VGCC function observed in our SCG experiments, these findings may reflect regulation at different levels and anatomical subdivisions of the sympathetic nervous system. Previous studies primarily assessed integrated systemic sympathetic activation, whereas our measurements examined local ganglionic NE accumulation and neuronal Ca^2+^ signaling under defined experimental conditions [6]. One possible explanation is that reduced VGCC-dependent signaling in the SCG represents a compensatory homeostatic adaptation during sustained central sympathetic drive. Autonomic ganglia can exhibit homeostatic plasticity [28], and NE has been shown to inhibit somatic Ca^2+^ currents and transmitter release in cultured SCG neurons through α2-adrenoceptor/G-protein signaling [29], providing a potential mechanism for inhibitory feedback during increased sympathetic activity. However, this study did not measure central sympathetic drive, so this adaptive explanation remains speculative. Furthermore, sympathetic outflow is organized in target-specific pathways [30,31], such that SCG pathways supplying head-and-neck structures, including the lacrimal gland, may not respond in parallel with sympathetic pathways innervating cardiovascular and renal targets [32,33,34]. Functional changes observed after direct exposure of isolated SCGs and cultured neurons also indicate that these effects can occur without intact central input. However, these experiments do not establish the route by which ICV administration produces SCG alterations.

The acute-exposure experiments provide additional context for interpreting VGCC dysfunction. Acute bath application of PM2.5 did not immediately suppress VGCC current under the conditions tested, whereas 24 h direct exposure attenuated depolarization-evoked Ca^2+^ responses. These observations do not support a readily detectable acute inhibitory action on VGCC currents and are compatible with effects involving cellular responses to prolonged exposure. Nevertheless, the different exposure conditions and functional readouts do not establish the time course of current suppression or distinguish specific channel regulation from broader cellular stress.

Depolarization-evoked Ca^2+^ responses were decreased in sympathetic neurons following PM2.5 exposure (Figure 3), and whole-cell recordings revealed reduced inward Ca^2+^ current density in neurons isolated after ICV administration, providing electrophysiological evidence of impaired VGCC function. VGCCs are multimeric complexes composed of a pore-forming α1 subunit and auxiliary subunits, including β and α2δ, as well as other regulatory proteins [35]. No significant changes were detected in the mRNA levels of Ca_v_1.2, Ca_v_1.3, Ca_v_2.1, Ca_v_2.2, and Ca_v_2.3 in whole-SCG tissue following ICV PM2.5 administration (Figure 4). These findings leave open the possibility of post-transcriptional and post-translational regulation. In particular, impaired trafficking or membrane insertion of α1 subunits could reduce surface density of functional channels at the plasma membrane without altering their transcript abundance [36,37]. The ubiquitin–proteasome system (UPS) is an important regulator of VGCC stability and membrane availability. RFP2-mediated ubiquitination promotes proteasomal degradation of Ca_v_1.2, whereas Nedd4-1 decreases Ca_v_1.2 surface expression by redirecting newly synthesized channels toward proteasomal and lysosomal degradation [38,39]. Parkin has also been shown to promote ubiquitin–proteasome-dependent degradation of Ca_v_2.2 and Ca_v_1.3, reducing channel protein abundance and current density [40,41]. These mechanisms provide candidate explanations for reduced VGCC currents despite no detectable changes in the examined transcripts. PM2.5 has been reported to activate aryl hydrocarbon receptor (AhR) signaling [42], and ligand-activated AhR can participate in ubiquitin-dependent protein degradation as part of an E3 ubiquitin ligase complex. However, whether PM2.5 engages an AhR–UPS pathway to alter VGCC surface abundance or degradation in sympathetic neurons remains unknown and was not directly examined in the present study. In addition, channel activity may be modified independently of protein abundance through phosphorylation, glycosylation, proteolytic processing, oxidative modification, or regulation by auxiliary subunits [38,43,44,45,46,47,48,49]. Thus, the observed reduction in VGCC current density may reflect altered channel trafficking, stability, or functional regulation that the present transcript measurements alone cannot resolve.

Although evidence directly linking PM2.5 exposure to VGCC surface abundance remains limited, PM2.5 exposure has been reported to decrease the expression of membrane-associated proteins, including the P2Y2 receptor and cystic fibrosis transmembrane conductance regulator (CFTR) [50]. In cardiomyocytes, PM2.5 exposure upregulated TRPC3, which interfered with L-type VGCC currents, contributed to abnormal Ca^2+^ handling, and increased arrhythmia susceptibility [51]. These findings indicate that PM2.5 can alter membrane-associated ion channel and receptor signaling, although it remains unclear whether similar mechanisms regulate VGCC surface expression in sympathetic neurons. Inflammatory mediators also regulate neuronal VGCCs [52,53]. In sympathetic neurons, TNF-α-induced suppression of N-type currents has been associated with reduced Ca_v_2.2 mRNA and membrane localization [53]. This provides a precedent for inflammatory modulation of VGCCs, although its accompanying transcriptional change differs from our findings. Because VGCC-mediated Ca^2+^ entry is a key trigger for vesicular NE secretion [13,14,54,55], reduced channel function is a plausible contributor to the lower extracellular NE accumulation observed here. However, recordings from neuronal somata do not directly establish impaired Ca^2+^ entry at release sites, and this functional link requires further testing.

Beyond neuron-intrinsic mechanisms, the SGC microenvironment may contribute to the observed impairment of VGCC function. SGCs closely envelop sympathetic neuronal cell bodies, forming functional neuron-glial units that help maintain the neuronal microenvironment [24,25,56]. Moreover, SGCs express Kir4.1, which plays an important role in regulating extracellular K^+^ homeostasis and neuronal excitability [15]. Changes in extracellular K^+^ can influence the neuronal resting membrane potential and, consequently, VGCC activation. Consistent with the possibility that PM2.5 can alter ion channel homeostasis, PM2.5-exposed rats showed reduced L-type VGCC (Ca_v_1.2) expression and increased inward-rectifier K^+^ channel (Kir2.1) expression in the myocardium [57]. In this study, increased GFAP expression and perineuronal GFAP immunoreactivity were accompanied by increased Kir4.1 protein levels in SCG tissue. However, total Kir4.1 abundance does not establish increased plasma-membrane localization, channel activity, or K^+^ buffering. The increase could reflect altered glial regulation or a compensatory response, and its functional significance remains unresolved.

SGCs can influence sympathetic neuron function through paracrine signaling. SGCs contribute to neuronal survival and synaptic activity through secreted factors [58].

Cytokine effects on VGCCs have been demonstrated in other neuronal systems: IL-1β suppresses Ca^2+^ currents through PKC-associated signaling in hippocampal neurons [59], while TNF-α modulates Ca^2+^ currents and their sensitivity to capsaicin in sensory neurons [60]. These findings support the plausibility of inflammatory regulation but do not establish SGC-derived cytokine signaling in the present SCG model. Moreover, sympathetic SGCs express purinergic receptors and ectonucleotidases that shape extracellular ATP and adenosine levels within the ganglion [61], and adenosine acting at A1 receptors inhibits N-type Ca^2+^ channels in SCG neurons through a pertussis toxin-sensitive G protein [62]. Gβγ-mediated inhibition of N-type channels and modulation of the release machinery provide additional routes through which extracellular signals can influence neurotransmission [63,64]. Together, these mechanisms suggest that changes in the SCG microenvironment could regulate sympathetic neuronal excitability and Ca^2+^-dependent neurotransmission. Consistent with this possibility, inflammatory stress has been shown to remodel Ca^2+^ signaling in rat sympathetic ganglia. LPS exposure increases GFAP expression while reducing Orai1 and STIM1 expression and decreasing SOCE [17]. This provides a precedent for concurrent glial and neuronal Ca^2+^ signaling changes during inflammation, although SOCE and VGCC-mediated entry represent distinct pathways. PM2.5-associated glial interactions in CNS models provide further context [65], but whether similar signaling operates within the SCG remains to be determined.

Nevertheless, inward Ca^2+^ current remained reduced under whole-cell recording conditions, in which VGCCs were directly activated using a voltage-ramp protocol and the extracellular environment was controlled. An immediate alteration of glial K^+^ buffering is therefore insufficient to explain the reduced currents recorded in isolated neurons. This does not exclude prior glial signaling that produces lasting neuronal changes. Disease-associated remodeling of neuronal ion-channel function has also been reported in peripheral autonomic efferent neurons [66]. In the present study, however, SGC reactivity and neuronal VGCC dysfunction should be interpreted as parallel observations rather than an established causal sequence. To determine their relationship, direct manipulation of SGC function or Kir4.1 expression, along with measurements of channel localization and activity, will be needed.

The present whole-cell recordings measured the composite VGCC current and therefore do not identify the specific channel subtype affected by PM2.5. The *I*–*V* relationship was dominated by high-voltage-activated currents, with no evident low-voltage-activated component under the recording condition used, consistent with previous reports characterizing VGCC subtypes in SCG neurons [67,68]. Thus, Ca_v_1 and Ca_v_2 channels represent the most relevant candidates for the PM2.5-sensitive current observed in our recordings. In SCG neurons, blockade of N-type VGCCs with ω-conotoxin GVIA has been reported to produce an inhibitory effect on NE secretion comparable to that of a non-selective Ca^2+^ channel blocker, whereas blockade of L-type VGCCs did not affect NE secretion under those experimental conditions [69]. These findings suggest that Ca_v_2.2 is an important candidate for a PM2.5-sensitive component involved in NE release. However, neither the *I*–*V* relationship nor the transcript measurements establish subtype-selective inhibition. Future studies using subtype-selective pharmacological approaches, such as ω-agatoxin IVA for Ca_v_2.1, ω-conotoxin GVIA for Ca_v_2.2, and dihydropyridines for Ca_v_1 channels, together with subtype-specific electrophysiological measurements, could quantify the PM2.5-sensitive fraction of each current and determine which VGCC subtype contributes to altered NE release.

An important consideration is the exposure route used in the present study. Although inhalation represents the physiologically relevant route of environmental exposure, accumulating evidence indicates that PM2.5 entering through respiratory and nasal pathways can reach the CNS [1,70]. ICV administration of PM2.5 provides a controlled approach for investigating central effects by bypassing pulmonary deposition and the initial barriers to CNS entry. However, this model does not fully reproduce real-world environmental exposure through inhalation, which may involve chronic or intermittent exposure, pulmonary responses, systemic inflammatory signaling, and different particle-distribution patterns. Moreover, the ICV experiments did not distinguish descending neural influences from circulating mediators or other routes that could contribute to SCG changes. Direct-exposure experiments demonstrate local susceptibility but do not establish that the SCG encounters comparable PM2.5 concentrations after ICV administration or environmental inhalation. Whether similar SCG dysfunction occurs following chronic or intermittent inhalation exposure, and how central, circulating, and peripheral mechanisms interact under such conditions remains to be determined.

Additional limitations concern the functional and molecular endpoints. We measured NE as accumulation in the culture medium over the exposure period rather than as stimulus-evoked exocytosis. This assay therefore does not distinguish reduced secretion from other processes affecting extracellular NE availability. Moreover, we assessed extracellular NE accumulation and neuronal VGCC currents in different exposure models, so we did not directly test their causal relationship. Unchanged TH expression does not establish preserved catecholamine synthesis rates. Similarly, targeted qPCR of selected α1 subunits in whole-SCG tissue cannot exclude changes in other transcripts or cell-type-specific responses. Transcriptomic profiling could identify broader stress, trafficking, and regulatory pathways, while measuring Hsp70, Hsp90, and other chaperones could clarify proteostatic responses not assessed here. These approaches would help distinguish among the candidate mechanisms raised by the present functional findings.

In conclusion, complementary exposure models revealed attenuated neuronal Ca^2+^ responses, reduced VGCC current density, and lower extracellular NE accumulation following PM2.5 exposure. ICV administration also induced hypothalamic inflammatory changes and SGC reactivity accompanied by increased Kir4.1 expression. These findings identify the SCG as a potential peripheral target of PM2.5 toxicity and support a possible contribution of impaired Ca^2+^-dependent secretion to sympathetic dysfunction. The mechanisms linking central exposure, neuronal channel impairment, and glial remodeling remain to be established.

## 4. Materials and Methods

### 4.1. Animals

This study used adult male Sprague–Dawley rats (8 weeks old). All rats were housed in individually ventilated cage (IVC) racks at a controlled temperature (22 ± 3 °C) and humidity (50 ± 10%) under lights from 6:00 to 18:00. All animal experiments were conducted according to the eighth edition of the Guide for the Care and Use of Laboratory Animals (NRC 2011), and all experimental protocols involving rats were approved by the Institutional Animal Care and Use Committee (IACUC) at Yonsei University Wonju College of Medicine (YWC-230605-1).

### 4.2. Primary Cell Isolation

Under anesthesia with isoflurane, a pair of superior cervical ganglia (SCG) were dissected out from male Sprague-Dawley rats and placed in cold Hank’s Balanced Salt Solution (HBSS, pH 7.4). SCGs were desheathed, cut into small pieces, and incubated with 1.2 mg/mL collagenase type D (Roche Diagnostics, Mannheim, Germany), 0.6 mg/mL trypsin (Worthington, Lakewood, NJ, USA), and 0.1 mg/mL DNase type I (Roche Diagnostics, Mannheim, Germany) in Earle’s balanced salt solution (EBSS, pH 7.4) in a 25-cm^2^ tissue culture flask. Then, the flasks were placed in a shaking water bath at 125 rpm at 37 °C for 60 min. After incubation, the ganglia were dispersed into single cells by vigorously shaking the flask. After centrifugation at 60× *g* for 6 min, the dissociated cells were resuspended in MEM (Gibco, Thermo Fisher Scientific, Waltham, MA, USA) containing 10% FBS (Gibco, Thermo Fisher Scientific, Waltham, MA, USA) and 1% penicillin-streptomycin (Hyclone, Logan, UT, USA). Cells were subsequently plated on culture dishes coated with poly-D-lysine (Sigma-Aldrich, St. Louis, MO, USA) and maintained at 37 °C, 5% CO_2_ until use. For direct treatment, isolated SCG neurons were treated with PM2.5 (100 μg/mL) or vehicle for 24 h.

### 4.3. Drug Treatment

Rats were anesthetized with intraperitoneal Avertin. Under anesthesia, rats were fixed in a stereotaxic apparatus (New Standard Stereotaxic, 51500, Stoelting Co., Wood Dale, IL, USA), and a hole was drilled (1.5 mm lateral and 0.0 mm anterior to bregma and ventrally 3.5 mm below the dura). Using a microinfusion pump (Legato 130, KD Scientific, Holliston, MA, USA) and a microsyringe (80330, Hamilton Company, Reno, NV, USA), a total dose of 300 μg of PM2.5 (SRM2786, NIST, Gaithersburg, MD, USA) or vehicle alone was administered intracerebroventricularly (ICV) at a rate of 1 μL/min. To prevent backflow, the needle was left in place for an additional 3 min before being slowly removed. After one week, rats were sacrificed.

### 4.4. Ex Vivo Experiments

Animals were decapitated, and the SCGs were immediately removed and placed in ice-cold MEM (Gibco, Thermo Fisher Scientific, Waltham, MA, USA). The SCGs were incubated in MEM supplemented with 10% FBS and 1% penicillin-streptomycin in 96-well plates (2 SCGs per well; 200 μL/well) for 24 h before treatment. The SCGs were then treated with PM2.5 (100 μg/mL) or vehicle for an additional 24 h. After treatment, we collected the culture medium, centrifuged it, and collected the supernatant for NE measurement. We immediately froze the supernatant and SCGs on dry ice and stored them at −60 °C until assay.

### 4.5. ELISA Assay

We measured NE levels by enzyme-linked immunosorbent assay (ELISA) (Eagle Biosciences, Amherst, NH, USA) following the manufacturer’s instructions.

### 4.6. Calcium Imaging

We measured intracellular Ca^2+^ as described previously [19]. Briefly, we loaded cells with 5 μM Fura-2, AM (Invitrogen, Carlsbad, CA, USA; F1221) in the dark at room temperature for 45 min. Fluorescence signals were measured at 2-s intervals (excitation, 340 nm/380 nm; emission, 520 nm). The 340 nm/380 nm fluorescence ratio reflects intracellular Ca^2+^. The normal physiological salt solution (PSS) used for bath perfusion contained (in mM) 135 NaCl, 5 KCl, 1 MgCl_2_, 2 CaCl_2_, 10 HEPES, and 5.5 glucose (pH 7.4). The high-potassium (HK) solution used to measure VGCCs contained (in mM) 60 NaCl, 80 KCl, 1 MgCl_2_, 2 CaCl_2_, 10 HEPES, and 5.5 glucose (pH 7.4).

### 4.7. Patch-Clamp Recording

We recorded currents using the whole-cell-dialyzed configuration of the patch-clamp technique as described previously [19]. We recorded whole-cell currents under voltage clamp using an EPC-9 patch-clamp amplifier (Heka Elektronik, Lambrecht, Germany). We coated the patch electrodes with silicone elastomer (Sylgard 184; Dow Corning, Midland, MI, USA), fire-polished them, and measured resistances of 2–3 MΩ when filled with pipette solution. The cell membrane capacitance and series resistance were compensated (>80%) electronically using an EPC-9 amplifier. Data acquisition was performed using the Pulse/Pulsefit (v8.50) software (Heka Elektronik, Lambrecht, Germany). All electrophysiological recordings were performed at room temperature (approximately 22–25 °C). The bath and pipette solutions contained (in mM) 140 CH_3_SO_3_H, 145 TEA-OH, 10 HEPES, 10 CaCl_2_, and 5 glucose (pH 7.4) and 120 NMG, 20 TEA-OH, 11 EGTA, 10 HEPES, 1 CaCl_2_, 4 MgATP, 0.3 Na_2_GTP, and 14 Tris Creatine PO_4_ (pH 7.2), respectively.

### 4.8. Real-Time PCR

Total RNA was obtained from cells or tissue. Complementary DNA was synthesized from 1 µg of the total RNA using the ReverTraAce^®^ qPCR RT Master Mix with gDNA Remover (FSQ-301, Toyobo, Osaka, Japan) according to the manufacturer’s instructions. We performed real-time PCR amplification on a QuantStudio 6 Flex Real-Time PCR System (Thermo Fisher Scientific, Waltham, MA, USA) using the QuantiTect SYBR Green PCR kit (Qiagen, Hilden, Germany). Thermal cycling conditions included 15 min polymerase activation at 95 °C, 40 cycles of 15 s at 95 °C for denaturation, and 60 s at 60 °C for annealing and extension. Table 1 lists the primer sequences used for quantitative real-time PCR.

### 4.9. Western Blot

Western blotting was performed as described previously [19]. For total protein extraction, cells and tissues were lysed with cold RIPA buffer (Thermo Scientific, Waltham, MA, USA), containing cOmplete ^TM^ EDTA-free protease inhibitor cocktail (Roche) and PhosSTOP (Roche). The lysates were centrifuged at 17,000× *g* for 20 min at 4 °C. We measured the protein concentration of each sample using the Pierce™ BCA Protein Assay Kit (Thermo Scientific, Waltham, MA, USA), following the manufacturer’s protocol. We denatured samples in Elpis Biotech Laemmli’s 5× sample buffer (Elpis Biotech, Daejeon, Republic of Korea) and loaded adjusted volumes onto an SDS-PAGE gel to ensure equal protein per well. The protein lysates were loaded onto SDS-PAGE gels and then transferred to a BioTrace^TM^ NT Nitrocellulose Transfer Membrane (Pall Corporation, Port Washington, NY, USA). We blocked the membrane with 5% skim milk for 1 h at room temperature, then incubated it with the primary antibody at 4 °C overnight. We incubated the membrane with a horseradish peroxidase (HRP)-conjugated secondary antibody against mouse or rabbit IgG for 1 h at room temperature. We visualized the bands with a ChemiDoc™ XRS+ imaging system (Bio-Rad, Hercules, CA, USA) using ECL™Select Western Blotting Detection Reagent (GE, Buc, France). Table 2 lists the primary antibodies used.

### 4.10. Immunostaining

We determined optimal dilutions and incubation times for all primary and secondary antibodies before experimental use. Rats were anesthetized with intraperitoneal Avertin and transcardially perfused with PBS, followed by 4% paraformaldehyde (PFA). We collected the SCGs, post-fixed them in 4% PFA for 24 h, and then cryoprotected them in 30% sucrose for at least 24 h. The tissues were then cryosectioned, mounted onto glass slides, and stored at −20 °C until use. Sections were first washed with PBS and permeabilized with 0.2% Triton X-100 for 10 min, followed by blocking with 3% normal goat serum for 1 h at room temperature. Primary antibodies were diluted appropriately and incubated overnight at 4 °C. The sections were then washed with PBS and incubated with the appropriate secondary antibodies for 1 h at room temperature. Sections were counterstained with 4′,6-diamidino-2-phenylindole dihydrochloride (DAPI) for 5 min to visualize the nuclei. We examined all staining results using confocal microscopy (LSM 800, ZEISS, Oberkochen, Germany) and analyzed them with ZEN 2.3 Lite (ZEISS).

### 4.11. Statistical Analysis

Statistical analyses were performed using Prism version 8.0 (GraphPad Software, San Diego, CA, USA). For comparisons between two groups, we used an unpaired two-tailed Student’s *t*-test when the data met the assumptions of parametric analysis. When the data did not meet the assumptions of normality, we used the two-tailed Mann–Whitney U test. Data are presented as mean ± SEM, and *p* ≤ 0.05 was considered statistically significant.

## Figures and Tables

**Table 1:** List of primers.

Form	Gene Name	FW (5′–3′)	RV (5′–3′)	Product Size (bp)
α1C (Cav 1.2α1)	*Cacna1c*	TGTCTGCTGCCTCCGAAGA	AGCTGACGTGGTTGCCAAA	66
α1D (Cav 1.3α1)	*Cacna1d*	CGTTTTGTTAAGCGATGCCAG	GCGCTACCCTTCCAGTTTACA	96
α1A (Cav 2.1α1)	*Cacna1a*	CCCAACGGGACCAAATGTC	GGATGTTCGAACTGAGTGATG	68
α1B (Cav 2.2α1)	*Cacna1b*	AAGGCGCTGCCCTACGT	GCCGATGATGGCGTAGATG	63
α1E (Cav 2.3α1)	*Cacna1e*	GCACTACATCTCTGAGCCCTATCTG	TCTCCTCCTCGCCACAGTCT	71
	*18S*	AACCCGTTGAACCCCATT	CCATCCAATCGGTAGTAGCG	149

**Table 2:** List of antibodies.

Target Antigen	Manufacturer	Catalog No.	Used Dilution	Applications
TH	Santa Cruz Biotechnology, Dallas, TX, USA	sc-25269	1:1000	WB, IF
GFAP	Abcam, Cambridge, UK	ab7260	1:1000	WB, IF
Synaptophysin	Proteintech, Rosemont, IL, USA	67864-1-PBS	1:1000	WB
IL-6	Santa Cruz Biotechnology, Dallas, TX, USA	sc-57315	1:300	WB
TNF-α	Cell Signaling Technology, Danvers, MA, USA	3707	1:1000	WB
Kir4.1	Alomone Labs, Jerusalem, Israel	APC 035	1:1000	WB
β-actin	Santa Cruz Biotechnology, Dallas, TX, USA	sc-47778	1:2000	WB
m-IgGκ BP-HRP	Santa Cruz, Dallas, TX, USA	sc-516102	1:3000	WB
Goat anti-Rabbit IgG Secondary Antibodies	Thermo Fisher Scientific, Waltham, MA, USA	G-21234	1:3000	WB

## Data Availability

The data used and/or analyzed during the current study are available from the corresponding author upon reasonable request.

## Supplementary Materials

The following supporting information can be downloaded at: https://www.mdpi.com/article/10.3390/ijms27209184/s1.

## Abbreviations

The following abbreviations are used in this manuscript:

CNS	Central nervous system
GFAP	Glial fibrillary acidic protein
ICV	Intracerebroventricular
IML	Intermediolateral nucleus
NE	Norepinephrine
PM2.5	Ambient fine particulate matter with an aerodynamic diameter ≤2.5 µm
SCG	Superior cervical ganglion
SGCs	Satellite glial cells
TH	Tyrosine hydroxylase
VGCC	Voltage-gated Ca^2+^ channel
