# Supplementary material for: PM2.5 Exposure Disrupts Sympathetic Neurotransmission by Impairing Voltage-Gated Ca2+ Channels and Remodeling Satellite Glia in the Superior Cervical Ganglion
Source: Int J Mol Sci. 2026 Oct 16;27(20):9184. doi: 10.3390/ijms27209184 (PMC13705502; doi:10.3390/ijms27209184)
Supplement: Supplementary file 1 [file ijms-27-09184-s001.zip › ijms-4591396-supplementary.pdf]

Supplementary Information

PM2.5 Exposure Disrupts Sympathetic Neurotransmission by Impairing Voltage-Gated Ca^{2+} Channels and Remodeling Satellite Glia in the Superior Cervical Ganglion

Phuong Anh Do [1,2,3,†], Huu Son Nguyen [1,2,3,4,†] and Seung-Kuy Cha [1,2,3,5,*]

[1] Department of Physiology, Yonsei University Wonju College of Medicine, Wonju, 26426, Republic of Korea;
phuonganh3110dkh@gmail.com (P.A.D.); son.nh3@vinuni.edu.vn (H.S.N.)
[2] Department of Global Medical Science, Yonsei University Wonju College of Medicine, Wonju, 26426, Republic of Korea
[3] Organelle Medicine Research Center, Yonsei University Wonju College of Medicine, Wonju, 26426 Republic of Korea
[4] Vinmec Research Institute of Stem Cell and Gene Technology, College of Health Sciences, VinUniversity, Hanoi, 100000, Vietnam
[5] Institute of Mitochondrial Medicine, Yonsei University Wonju College of Medicine, Wonju, 26426, Republic of Korea
* Correspondence: skcha@yonsei.ac.kr; Tel.: +82-33-741-0295
† These authors contributed equally to this work.

Table of Contents

Supplementary Figure S1. Experimental procedure for intracerebroventricular administration of PM2.5.................. 3

Supplementary Figure S2. Whole-cell patch-clamp recording of voltage-gated Ca^{2+} channel currents in SCG neurons following PM2.5 exposure... 4

Supplementary Figure S3. Effects of PM2.5 exposure on voltage-gated Ca^{2+} channel currents in SCG neurons.. 5

Supplementary Materials and Methods 6

Patch-clamp recording 6

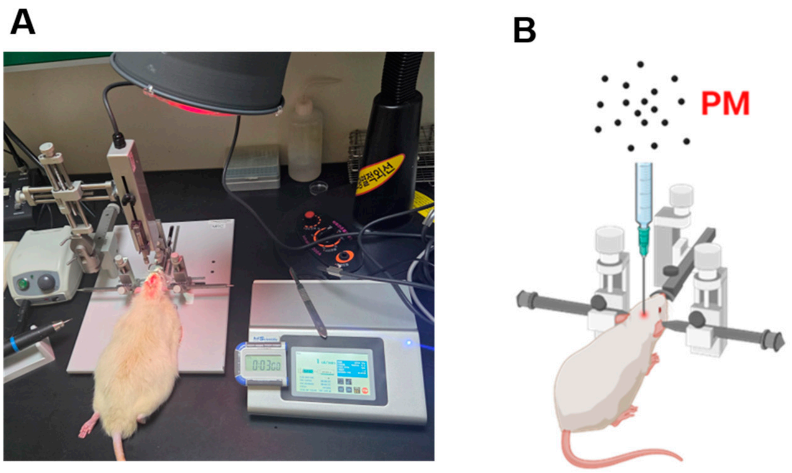

Supplementary Figure S1. Experimental procedure for intracerebroventricular administration of PM2.5. (A) Representative photograph showing a rat positioned in a stereotaxic apparatus for intracerebroventricular (ICV) injection. (B) Schematic representation of stereotaxic administration of PM2.5 into the cerebral ventricle.

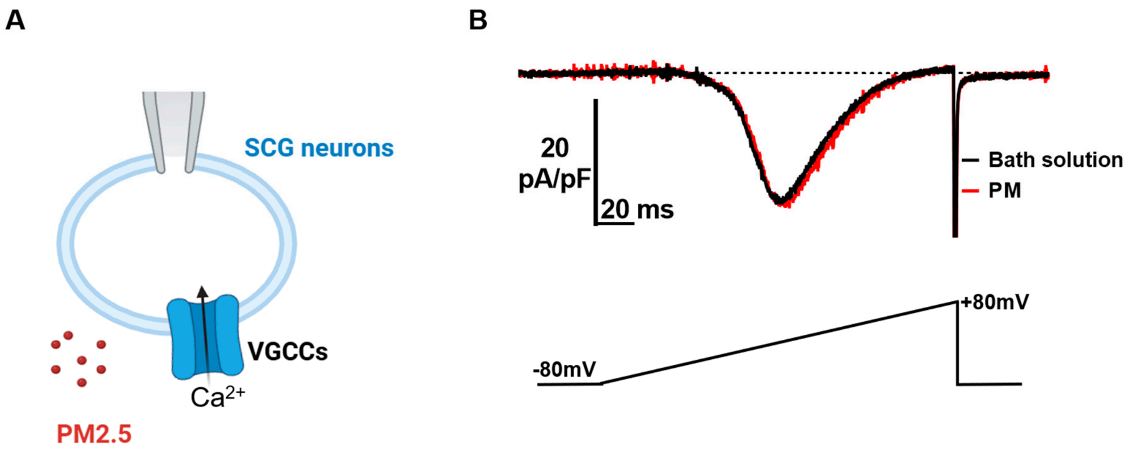

Supplementary Figure S2. Whole-cell 2. channel currents in SCG neurons following PM2.5 exposure. (A) Schematic illustration of whole-cell patch-clamp recording of VGCC currents in SCG neurons. (B) VGCC current traces from a single SCG neuron, recorded before and after acute bath application of PM2.5, elicited using a voltage-ramp protocol from -80 to +80 mV. Black and red traces represent recordings before and after PM2.5 application, respectively. VGCC, voltage-gated Ca^{2+} channel; SCG, superior cervical ganglion.

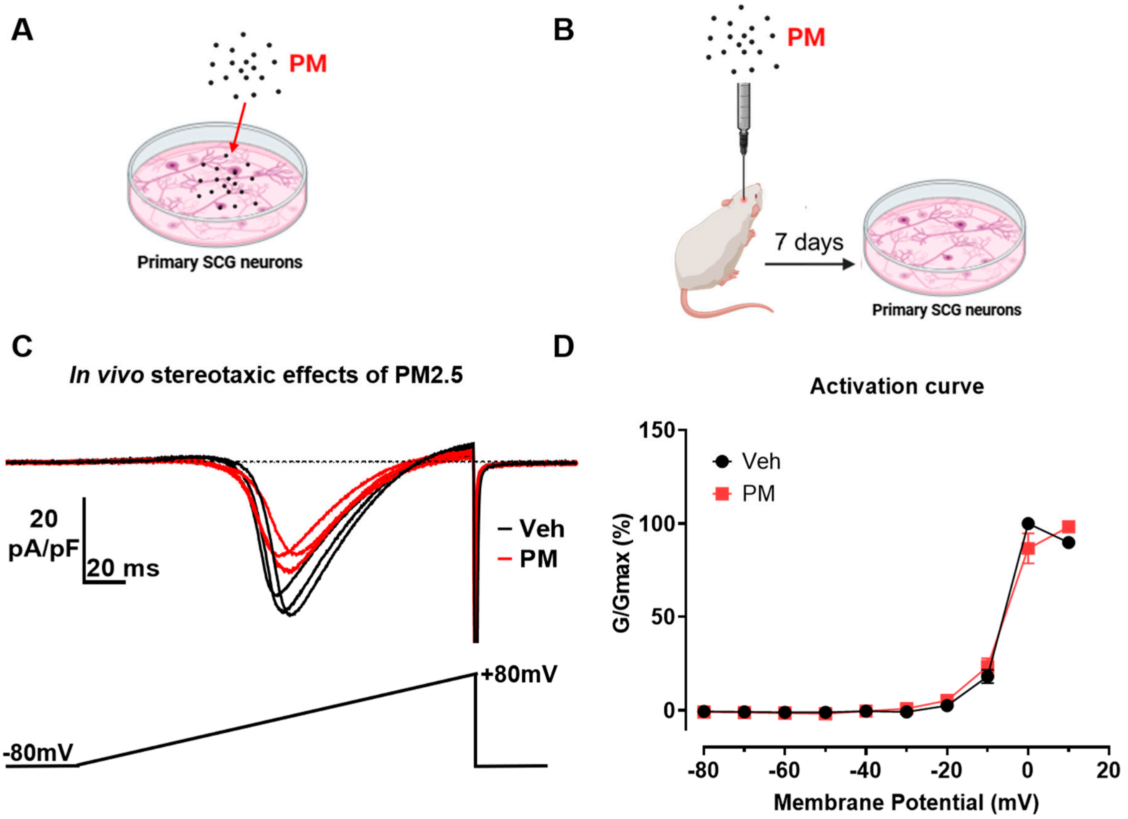

Supplementary Figure S3. Effects of PM2.5 exposure on voltage-gated Ca^{2+} channel currents in SCG neurons. (A) Schematic illustration of direct PM2.5 exposure to primary SCG neurons. (B) Schematic illustration of *in vivo* PM2.5 administration followed by SCG collection and isolation of primary SCG neurons for culture. (C) Individual VGCC current traces from all three SCG neurons per group included in the analysis shown in Figure 3E and F, elicited using a voltage-ramp protocol from -80 to +80 mV. Black and red traces represent the vehicle or PM2.5 groups, respectively. (D) Conductance-voltage relationship of VGCC currents in SCG neurons isolated from vehicle- or PM2.5-treated rats. Conductance is expressed as normalized conductance (G/Gmax, %). VGCC, voltage-gated Ca^{2+} channel; SCG, superior cervical ganglion.

Supplementary Materials and Methods

Patch-clamp recording for acute PM2.5 application

SCG neurons were isolated from 2-month-old rats and cultured on poly-D-lysine-coated coverslips in MEM (Gibco, Thermo Fisher Scientific, Waltham, MA, USA) containing 10% FBS (Gibco, Thermo Fisher Scientific, Waltham, MA, USA) and 1% penicillin-streptomycin (Hyclone, Logan, UT, USA) at 37 °C, 5% CO_2. After three hours of culture, whole-cell patch-clamp recordings were performed as described in the main manuscript.

The bath and pipette solutions contained (in mM) 140 CH_3SO_3H, 145 TEA-OH, 10 HEPES, 10 $CaCl_2$, and 5 glucose (pH 7.4) and 120 NMG, 20 TEA-OH, 11 EGTA, 10 HEPES, 1 $CaCl_2$, 4 MgATP, 0.3 Na_2GTP, and 14 Tris Creatine PO_4 (pH 7.2), respectively.

After establishing the whole-cell configuration at a holding potential of -80 mV using a pipette (2–3 MΩ), VGCC currents were initially recorded under normal conditions (bath solution only). Currents were elicited using a voltage-ramp protocol from -80 to +80 mV in 300 ms, followed by construction of the current-voltage (*I-V*) relationship. PM2.5 were then directly applied to the bath solution at a final concentration of 100 μg/mL. During acute PM2.5 application, VGCC currents were recorded using the same voltage-ramp protocol, followed by measurement of *I-V* relationship under PM2.5-exposed conditions.
